# Atomic Force Microscopy-Based Nanoscopy of Chondrogenically Differentiating Human Adipose-Derived Stem Cells: Nanostructure and Integrin β1 Expression

**DOI:** 10.1186/s11671-018-2722-z

**Published:** 2018-10-23

**Authors:** Jie Yang, Ming-Tang He, Xun Huang, Qiu-Shi Wang, Jiang Pi, Hua-Jun Wang, Ali Hasan Rahhal, Si-Min Luo, Zhen-Gang Zha

**Affiliations:** 1 Institute of Orthopedic Diseases and Center for Joint Surgery and Sports Medicine, the First Affiliated Hospital, Jinan University, Guangzhou, 510630 People’s Republic of China; 2 Longgang Orthopedics Hospital of Shenzhen, Shenzhen, People’s Republic of China; 3 Department of Materials Science and Engineering, Jinan University, Guangzhou, People’s Republic of China; 4 Department of Microbiology and Immunology, University of Illinois at Chicago, Chicago, IL USA

**Keywords:** Atomic force microscopy, Chondrogenic differentiation, Integrin β1, Human adipose-derived stem cells, β-catenin/SOX signaling pathway

## Abstract

Integrin β1 is known to be involved in differentiation, migration, proliferation, wound repair, tissue development, and organogenesis. In order to analyze the binding probability between integrin β1 ligand and cluster of differentiation 29 (CD29) receptors, atomic force microscopy (AFM) was used to detect native integrin β1-coupled receptors on the surface of human adipose-derived stem cells (hADSc). The binding probability of integrin β1 ligand–receptor interaction was probed by integrin β1-functionalized tips on hADSc during early chondrogenic differentiation at the two-dimensional cell culture level. Cell morphology and ultrastructure of hADSc were measured by AFM, which demonstrated that long spindled cells became polygonal cells with decreased length/width ratios and increased roughness during chondrogenic induction. The binding of integrin β1 ligand and CD29 receptors was detected by β1-functionalized tips for living hADSc. A total of 1200 curves were recorded at 0, 6, and 12 days of chondrogenic induction. Average rupture forces were, respectively, 61.8 ± 22.2 pN, 60 ± 20.2 pN, and 67.2 ± 22.0 pN. Rupture events were 19.58 ± 1.74%, 28.03 ± 2.05%, and 33.4 ± 1.89%, respectively, which demonstrated that binding probability was increased between integrin β1 ligand and receptors on the surface of hADSc during chondrogenic induction. Integrin β1 and the β-catenin/SOX signaling pathway were correlated during chondrogenic differentiation. The results of this investigation imply that AFM offers kinetic and visual insight into the changes in integrin β1 ligand–CD29 receptor binding on hADSc during chondrogenesis. Changes in cellular morphology, membrane ultrastructure, and the probability of ligand–transmembrane receptor binding were demonstrated to be useful markers for evaluation of the chondrogenic differentiation process.

**Electronic supplementary material:**

The online version of this article (10.1186/s11671-018-2722-z) contains supplementary material, which is available to authorized users.

## Background

Osteoarthritis (OA) is a common, degenerative-joint disease of the elderly [1], with degenerative OA characterized by progressive destruction of the articular cartilage. Cartilage is highly organized without blood vessels, nerves, or lymphatic tissue [2]. The extracellular matrix (ECM) is mainly comprised of collagen II and glycoprotein, and it is very important for cartilage homeostasis. Since cartilage is avascular, its capacity for self-renewal is limited. Although OA treatments (both surgical and non-surgical) can quickly relieve OA patient symptoms, especially pain, they cannot restore normal structure and function to the joint cartilage [3]. In the future, treatment will likely include tissue engineering with stem cells and scaffolds to repair defects and degenerative joint cartilage [4]. Mesenchymal stem cells are multipotent stromal cells that have osteogenic, adipogenic, chondrogenic, and myogenic potential, depending upon growth factor combinations [5]. Analysis of mesenchymal stem cell differentiation has shown Wnt/β-catenin, mammalian target of rapamycin (mTOR), phosphoinositide 3-kinase (PI3K), and other pathways to play important roles in differentiation [6–8]. However, the underlying mechanism by which chondrogenic differentiation is induced remains elusive. This is particularly true for the mechanism by which extracellular signals activate intracellular signaling pathways. We have found integrin β1 to undergo change during chondrogenic differentiation. Therefore, we hypothesized that integrin β1 may play an important role in human adipose-derived stem cell (hADSc) chondrogenic differentiation due to its involvement in various tissue-differentiation signaling pathways. In this investigation, the focus was on the Wnt/β-catenin signaling pathway.

Numerous studies have shown interactions between cells and the extracellular environment are regulated by transmembrane proteins, in particular, integrin family members [9]. Integrins are composed of heterodimeric-transmembrane glycoproteins of non-covalently bonded α and β chains [10]. Theoretically, there are 64 known integrins of which only 24 have been found. Integrins play vital roles in cell–cell adhesion, ECM-cell adhesion, cell signaling, and the organization of the actin cytoskeleton [11]. The ECM plays an important role in tissue homeostasis, and that the ECM regulates integrins. Integrins mediate lots of fundamental processes including cell adhesion, migration, proliferation, differentiation, cell death, wound repair, tissue development, and organogenesis. During mesenchymal stem cell chondrogenic differentiation, the expression of integrin β1 is connected to the SOX signaling pathway and to collagen II. The focus of this investigation was on the integrin β1 dimer since it is the most prominent β dimer among cartilage heterodimers and is known to interact with many different α dimers [12]. The cluster of differentiation 29 (CD29) is an integrin β1 subunit associated with very late antigen receptors, expressed on nearly all cells and tissue types.

Here, atomic force microscopy (AFM) was used to help us measure the changes during hADSc chondrogenic differentiation. As a very high-resolution type of scanning probe microscopy, AFM has provided a new opportunity to detect morphology and cellular membrane for single cells in fluid at nanoscale. Meanwhile, the system of single-molecule force spectroscopy (SMFS) combined by atomic force microscopy (AFM) was used to measure ligand–receptor binding on the living cells. The system of SMFS was more sensitive to the changes of receptors in cellular membrane, and the images of binding force were visualized. In this work, integrin β1 ligand–receptor binding was probed by integrin β1-functionalized AFM tips. Applying AFM, chondrogenic differentiation was found to change hADSc cell shape and to increase cellular roughness. This application provided a method by which to assess chondrogenic differentiation by direct measurement of integrin β1 ligand–receptor interactions and cell surface ultrastructure alteration, improving cell surface investigation and screening at a visualized way. Chondrogenic differentiation changes membrane composition and structure, as well as intracellular cytoskeletal interactions. These changes in cellular morphology, ultrastructure, and ligand–transmembrane receptor binding serve as useful markers for the evaluation of chondrogenic-differentiation mechanisms.

## Methods

### Cell Culture and Reagents

For this investigation, cells were isolated from three surgical patients (mean age 20 years) as described previously [13]. Informed consents were obtained from all patients. Ethics approval for this study was obtained from the First Affiliated Hospital of Jinan University (supplement form). Cells were maintained in basal medium, which included low-glucose Dulbecco’s Modified Eagle’s Medium (DMEM, Life Technologies, CA, USA) supplemented with 10% heat-inactivated fetal bovine serum (FBS, Life Technologies, CA, USA), 100 units/ml penicillin (Life Technologies, CA, USA), 100 μg/ml streptomycin (Life Technologies, CA, USA), 0.11 mg/ml sodium pyruvate (Life Technologies, CA, USA), and L-glutamine (Life Technologies, CA, USA). Cells were maintained at 37 °C in a humidified incubator containing 5% CO_2_ with medium changed every 3 days.

### In Vitro Differentiation

For chondrogenic induction, fourth- to eighth-passage hADSc were seeded at a high-cell density (2 × 105/10 ml) and cultured in chondrogenic medium containing DMEM/F12 supplemented with 1% FBS, 1% Insulin-Transferrin-Selenium (ITS) + supplement (Cyagen, Guangzhou, China), 10 ng/ml transforming growth factor-beta1 (TGF-β1) (Peprotech, Rocky Hill, New Jersey, USA), 100 ng/ml insulin-like growth factors-1 (IGF-1) (Peprotech, Peprotech, Rocky Hill, New Jersey, USA), 10-7 M dexamethasone (Sigma, St. Louis, MO, USA), and 50 μg/ml ascorbic acid (Sigma, St. Louis, MO, USA). The medium was changed every 2 days with TGF-β1 and IGF-1 freshly added. Chondrogenesis was assessed by alcian blue and toluidine blue staining.

To induce osteogenic and adipogenic differentiation, fourth- to eighth-passage cells were treated with the osteogenic and adipogenic medium for 2 weeks, respectively. Osteogenic medium consisted of DMEM supplemented with 10-7 M dexamethasone (Sigma, St. Louis, MO, USA), 50 μg/ml ascorbic acid (Sigma, St. Louis, MO, USA), and 10 mmol/l β-glycerol phosphate (Sigma, St. Louis, MO, USA). Osteogenesis was assessed by alizarin red staining.

Adipogenic medium consisted of DMEM supplemented with 0.5 mmol/l 3-isobutyl-1-methylxanthine (IBMX) (Sigma, St. Louis, MO, USA), 1 μmol/l hydrocortisone (Sigma, St. Louis, MO, USA), 0.1 mmol/l indomethacin (Sigma, St. Louis, MO, USA). Adipogenic differentiation was evaluated by Oil Red O staining.

### Identification of hADSc Surface Antigens by Flow Cytometry

The hADSCs were digested with trypsin and then rinsed twice with DMEM, prior to re-suspension at a cell density of 2 × 10^7^ cells/ml. The cell suspension (50 μl; 1 × 10^6^ cells) was added to 1.5 ml epoxy epoxide tubes and then incubated with anti-CD34, anti-CD44, anti-CD45, anti-CD73, anti-CD90, anti-CD106, anti-HLA-DR, and anti-CD105 antibodies for 20 min at 37 °C in the dark. The anti-CD34, anti-CD44, and anti-CD45 were obtained from CST (Beverly, MA, USA); other antibodies were obtained from Abcam (Cambridge, MA, USA). Then, the cell suspension was centrifuged at × 500*g* for 5 min, followed by removal of the supernatant and resuspension of the cells in 200 μl of Stain Buffer. All steps were repeated twice prior to analysis by flow cytometry.

### Immunoblotting Analysis (IB)

Cells were collected for immunoblotting as described previously [14]. The primary antibodies used were anti-β-catenin (ab32572), anti-integrin β1 (ab30394), and anti-collagen II (ab34712), obtained from Abcam (Cambridge, MA, USA). Anti-β-actin (8H10D10, 1:2000), anti-GSK-3β (27C10, 1:1000), and anti-SOX (92G2, 1:1000) were obtained from Cell Signaling Technology (CST, Beverly, MA, USA). Secondary HRP-conjugated antibodies (1:1000–1:3000) were purchased from CST.

### Immunofluorescence

For chondrogenic differentiation, cells were treated for 0, 6, and 12 days, digested, and cultured on the glass in 24-well plates (Costar353047, Corning, New York, USA) for 24 h. Cells were washed twice with ice-cold phosphate buffer solution (PBS), fixed with 4% paraformaldehyde for 15 min at room temperature. After blocking, cells were incubated with the primary antibody reactive with integrin β1 for 1 h, followed by incubation for 1 h in the dark with Alexa Fluor 488-labeled anti-mouse IgG (H + L) (CST #4408, MA, USA), 4′,6-diamidino-2-phenylindole (DAPI, Sigma, MO, USA). For phalloidin staining, after blocking, cells were permeabilized with 0.2% Triton X-100 for 30 min, then the cells were incubated with DAPI and phalloidin-Alexa Flour 573 (Life technologies, CA, USA) for 1 h. After washing three times, subcellular localization of integrin β1 and the change of filamentous actin (F-actin) were assessed during cartilage differentiation with a Laser Scan Confocal Microscope (ZEISS, LSM 700, Oberkochen, Germany).

### AFM Tips Preparation

The Si3N4 tips (DNP-10, Bruker Corp) with a spring constant (0.06 N/m) was chemically modified by the anti-CD29 antibody as follows [15]. The tips were cleaned with acetone, ultraviolet light, and piranha solution (H_2_SO_4_:H_2_O_2_ = 3:1, *v*/*v*) for different times (5 min, 30 min, and 10 min). After thorough rinsing with purified water, tips were formed by incubation with a solution of 1% 3-APTES (Sigma, St. Louis, MO, USA) in ethanol for 30 min. The tips were washed with ultrapure water three times and treated with 2.5% glutaraldehyde (Sigma, St. Louis, MO, USA) solution for 1 h. Superfluous glutaraldehyde was washed three times with water. Finally, the tips were inserted in an anti-integrin β1 solution (1 mg/ml) and incubated overnight at 4 °C. The modified probes were washed with PBS before experiments.

### AFM Measurements

AFM (Bioscope Catalyst, Bruker, USA) was used to investigate the hADSc morphology and ultrastructural changes during chondrogenic differentiation. The exact force constant of AFM tips was measured in PBS. To assess morphology and ultrastructure, cells were washed with PBS several times. Then, 4% paraformaldehyde solution was added into a 3.5-cm^2^ culture dish for 15 min. After the cells were washed with PBS, cells were stored in PBS at 4 °C till used. The spring constant of tips ranged from 4.2 to 5.8 N/m in contact mode. Morphology and ultrastructural images of hADSc were taken in PBS at room temperature by AFM. Ultrastructure images surrounding nuclei of hADSc were attained in contact mode. Nanoscope analysis software was used to evaluate cell surface ultrastructure for more than 15 different 10 × 10 μm images for at least 15 different cells in (day 0, 6, 12) groups. Binding force between the integrin β1-modified AFM tips and the CD29 receptors of living hADSc was analyzed during different chondrogenic periods (0, 6, and 12 days). The binding force was measured in the approach-retract mode of AFM system (Bioscope Catalyst, Bruker, USA). To study the integrin β1–living cell separation events, the integrin β1 antibody-modified tips were used at approach-retract velocities of 500 nm/s. The force constant of functionalized tips was 0.058 ± 0.006 N/m. The threshold force on cells was 800 pN. The anti-integrin β1 antibody (100 μg/ml) was added to cells for 30 min before force measurement experiments. Integrin β1 blocking and bare probes were also used as controls to detect the unspecific rupture force between integrin β1 antibody-modified tips and cells. For quantification of integrin β1 ligand−receptor binding probability, specific interaction force curves were measured by integrin β1 antibody-functionalized probes. More than 400 force curves were measured in a single experiment with results summarized from at least three independent experiments. Thus, approximately 1200 original force–distance curves in each comparison experiment were acquired from 30–40 different cells using the instrument’s Nanoscope analysis software. By averaging force values for at least three independent experiments, the effect of chondrogenic induction on the interaction force between integrin β1 ligand and CD29 receptors on the cell surface was determined.

### Reverse Transcription and Real-time PCR

TRIzol® Plus RNA Purification Kits (Life Technologies, CA, USA) were used, and 1 μg of RNA was reverse transcribed to cDNA using a High Capacity cDNA Reverse Transcription Kit (Invitrogen) according to the manufacturer’s protocol with minor modification. Integrin β1 and GAPDH were quantified using qRT-PCR with gene specific primers: 5′-TGGAGGAAATGGTGTTTGC-3′ (integrin β1-sense) and 5′-CGTTGCTGGCTTCACAAGTA-3′ (integrin β1-antisense); 5′-CTGACTTCAACAGCGACACC-3′ (GAPDH-sense) and 5′-CCCTGTTGCTGTAGCCAAAT-3′ (GAPDH-antisense). For real-time PCR, Step One Real-Time PCR (Applied Biosystems) was performed using Fast SYBR@GREEN Master Mix (Life Technologies, CA, USA). Target gene expression was normalized to GAPDH as an internal standard and calculated using the comparative 2-ΔΔCT method. Each assay was conducted in triplicate.

### Statistical Analysis

All experiments were performed at least three times, with data expressed as the mean ± standard deviation (SD). Comparison between two groups was conducted by *t* test. Significant differences among group means were determined by one-way ANOVA analysis, followed by Bonferroni and Tamhane’s T2’s test (equal variances were not assumed). Values of *p* < 0.05 were considered statistically significant.

## Results and Discussion

### Assessment of hADSc

Mesenchymal stem cells are multipotent stromal cells that have osteogenic, adipogenic, chondrogenic, and myogenic potential. There are two principal means by which to identify hADSc, cell surface CD markers and the capacity to differentiate [16]. As shown in Additional file 1: Figure S1 and Additional file 2: Figure S2, the derived cells were hADSc. Then, cell proliferation of passage 3 hADSc was determined by MTT assay (Additional file 3: Figure S3).

### Induced Morphology and Surface Ultrastructure Changes during hADSc Chondrogenesis

**Fig. 1:**
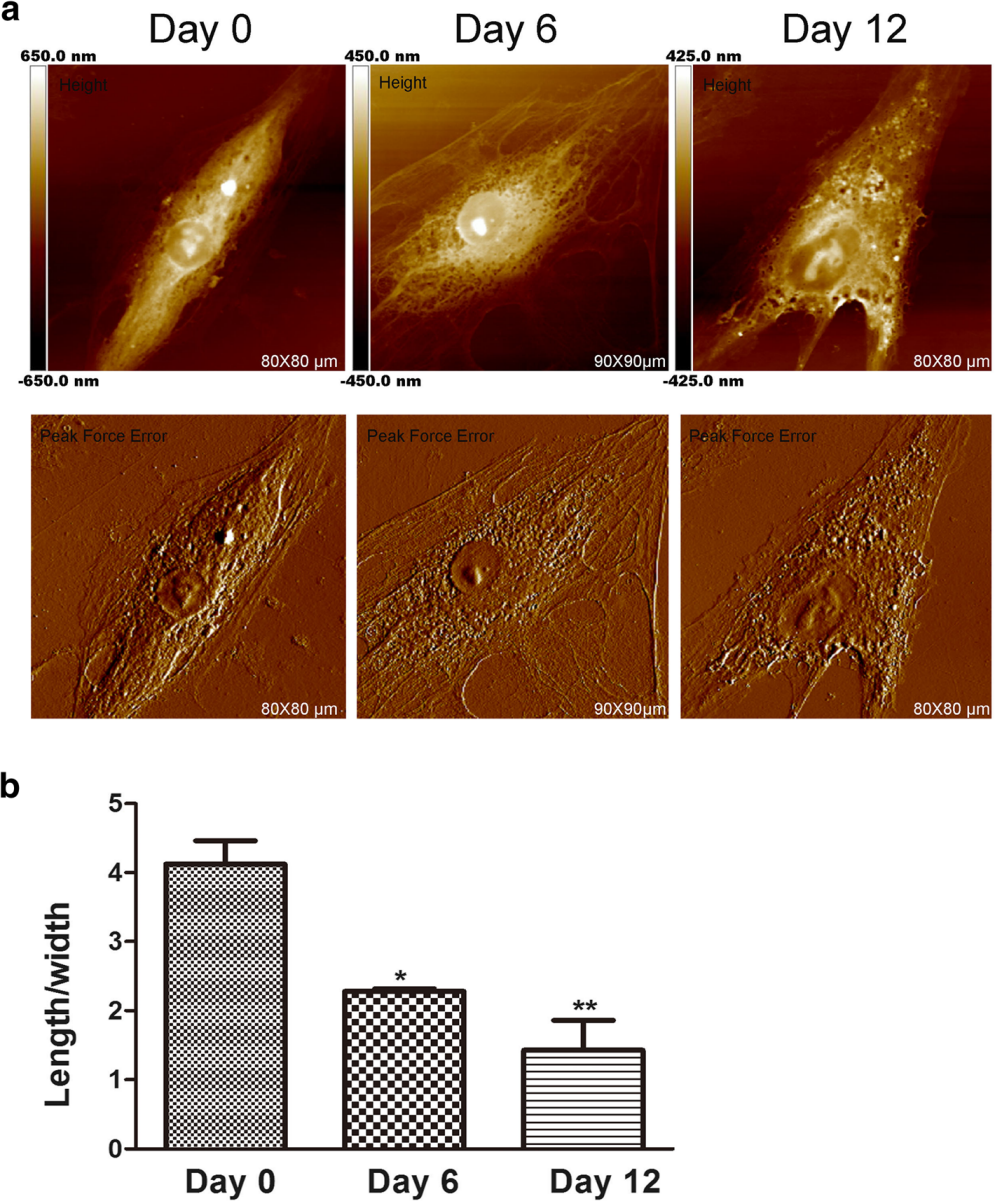
Characteristics of hADSc morphology during chondrogenesis. **a** Morphological images of whole hADSc were obtained at 0, 6, and 12 days of chondrogenic differentiation. Images were analyzed by a Height and Peak Force Error Image Model by Nanoscope. **b** The average length/width ratio of cells was measured following chondrogenic differentiation treatment at 0, 6, and 12 days. *p < 0.05, **p < 0.01

**Fig. 2:**
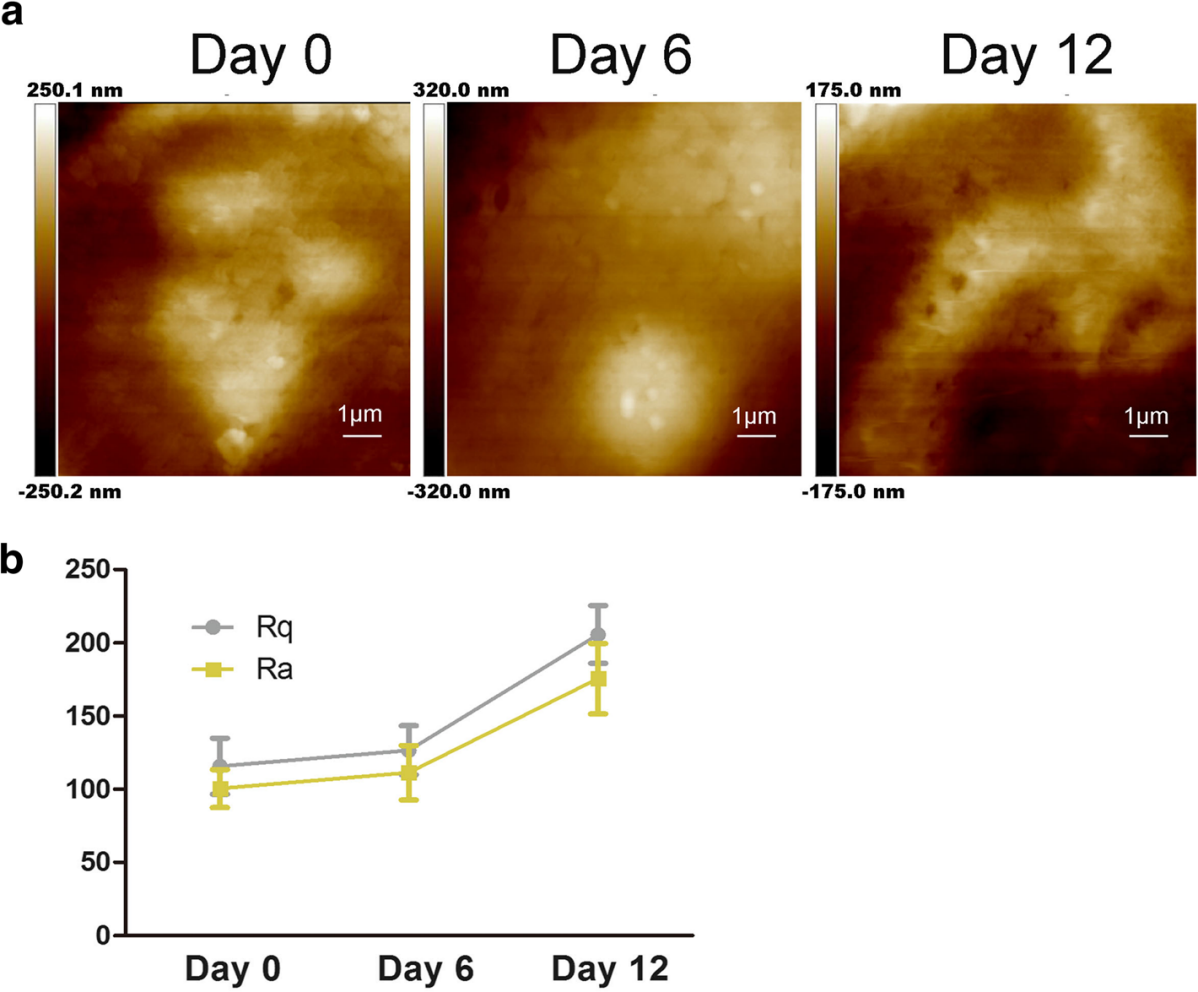
Characteristics of hADSc membrane ultrastructure during chondrogenic differentiation. **a** Changes in cell membrane ultrastructure were assessed after chondrogenic differentiation for 0, 6, and 12 days. **b** The surface roughness parameters Ra and Rq of cells were measured during chondrogenic induction of hADSc for 0, 6, and 12 days

AFM is always used to detect cell morphology and ultrastructure at nanoscale [17]. The shape of a cell relates to its specialized cell function and to tissue organization. In some cancer research, AFM can be used as a high-imaging technique to analyze morphological changes for the evaluation of drug effects. Further, mesenchymal stem cell shape is changed during chondrogenic induction [18]. While changes of cell shape appear to be necessary for differentiation, little is known about whether cell morphology affects earlier developmental stages of mesenchymal stem cell differentiation. Therefore, morphology and membrane ultrastructure changes during hADSc chondrogenesis were evaluated by AFM, since these changes are important [19] and can directly influence the function of cells [20]. Surface morphology and ultrafine structure of hADSc were investigated during chondrogenic differentiation for differing time periods (Fig. 1 and Fig. 2). The morphology and surface ultrastructure were obviously different in each comparison group. On day 0, cells had an elongated spindle shape with a relatively smooth surface. Cell membrane architecture was homogeneous. After chondrogenic induction, at days 6 and 12, significant cell morphology changes were observed. Most of the cells gradually shrank into a polygonal shape (Fig. 1a) with a decrease in average cell length/width ratio during chondrogenic differentiation (Fig. 1b). Numerous studies show the changes of cell morphology are consistent with the cytoskeleton of cells [21]. We also found the cytoskeleton changes during chondrogenic differentiation, which was explained in the latter results.

As shown in Fig. 2a, cell membrane ultrastructure also changed; particles became enlarged and were heterogeneous. Previous studies demonstrated Ra and Rq were the makers of the roughness value to evaluate the change in differently treated cell membranes [22]. Rq is about root-mean-squared roughness, $\mathrm{Rq}=\sqrt{\frac{\sum_{t-1}^N{\left( Zn-\overline{Z}\right)}^2}{N-1}}$; $\mathrm{Rq}=\sqrt{\frac{\sum_{\mathrm{t}-1}^{\mathrm{N}}{\left(\mathrm{Zn}-\overline{\mathrm{Z}}\right)}^2}{\mathrm{N}-1}};$ Ra is about average roughness, $\mathrm{Ra}=\frac{1}{N}{\sum}_{t-1}^N1\mid Zi-\overline{Z}\mid$. To get the roughness, the scan size is 10 μm × 10 μm. As shown in Fig. 2b, both the Ra and Rq of two different areas increased during chondrogenesis of hADSc. The Ra and Rq values of the cells at day 0 were low, indicating a smooth surface (Fig. 2b). The values for Ra and Rq concurrently increased with chondrogenic differentiation, showing greater heterogeneity and rougher on cell surfaces (Fig. 2a). Based on observed changes, chondrogenic differentiation resulted in cell morphology and cell height/width ratio changes (Fig. 1a, b). There are studies showing that ECM could regulate cell adhesion by regulating integrins [11]. Therefore, increased roughness values suggested changes in ECM and the ultrastructure of the cell membrane during chondrogenesis. These data demonstrate chondrogenic differentiation to affect cell morphology, the ECM, and cell membrane structure.

### Cytoskeletal Changes during Chondrogenic Induction of hADSc

**Fig. 3:**
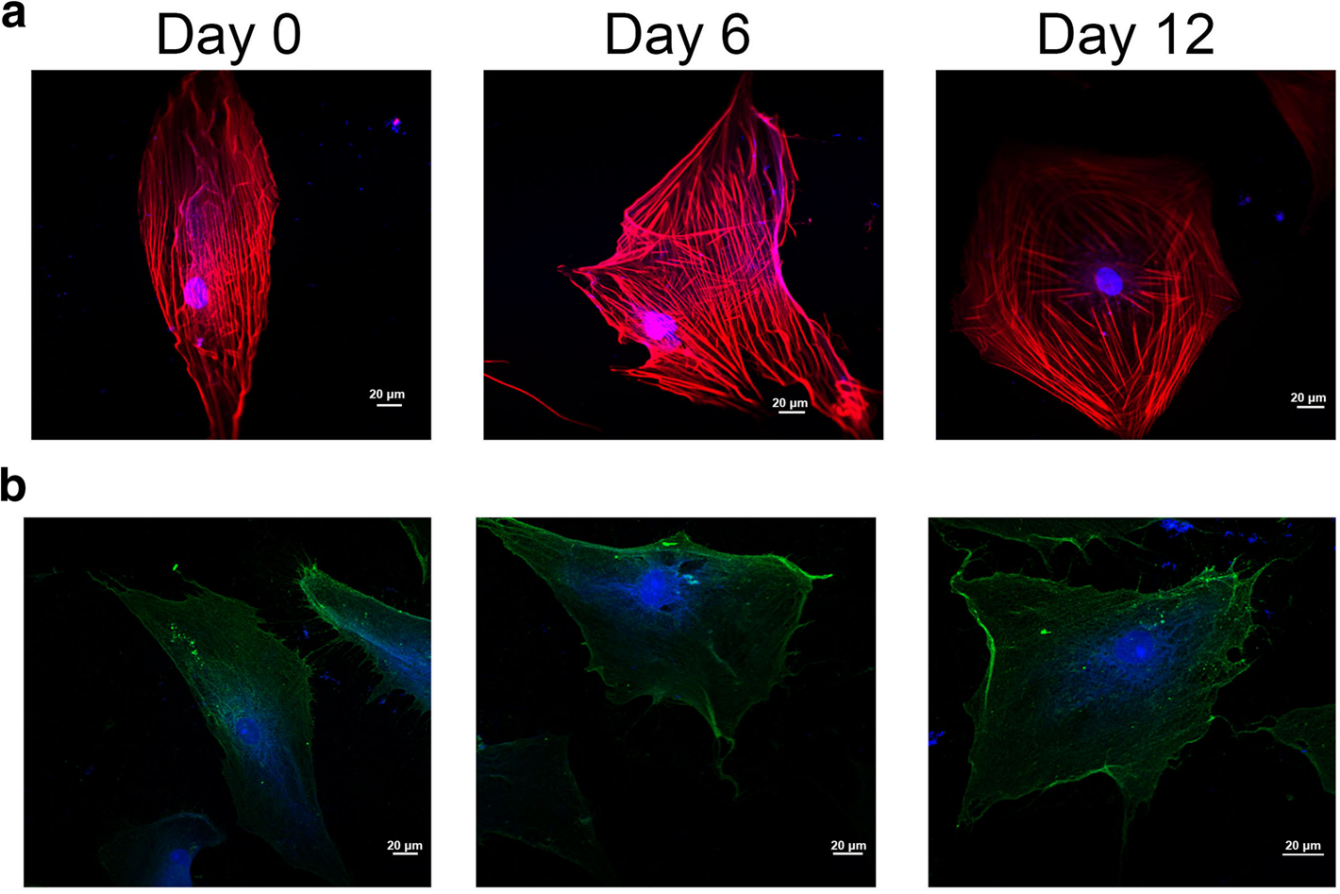
Organization of the cytoskeleton and location of integrin β1 on chondrogenically differentiating hADSc. **a** Changes in cytoskeleton were detected during chondrogenesis of hADSc by confocal microscopy. **b** The location of integrin β1 was measured during chondrogenic differentiation by confocal microscopy. Cytoskeleton and nucleus were stained with F-actin and DAPI, respectively. The red and blue fluorescence signals respectively indicate F-actin and DAPI

During stem cell differentiation, cell morphology and membrane structural changes are related to the cytoskeleton of the cell, subsequent to the development of lineage-specific cellular characteristics [21]. As shown in Fig. 3a, the red and blue fluorescence signals respectively indicate F-actin and DAPI. The cell cytoskeleton changed greatly during chondrogenic induction in Fig. 3a. On the one hand, the microfilaments of cytoskeleton went along the long cell axis at day 0 group, while cytoskeleton microfilaments spread in a radial array when hADSc were treated with chondrogenic differentiation for 12 days. On the other hand, the distribution of cell microfilaments was homogeneous at day 0 group but the microfilaments were mainly distributed at the periphery of hADSc treated with chondrogenic differentiation for 12 days.

### Chondrogenic Differentiation Changed the Binding Probability of Integrin β1 to Receptors on hADSc

**Fig. 4:**
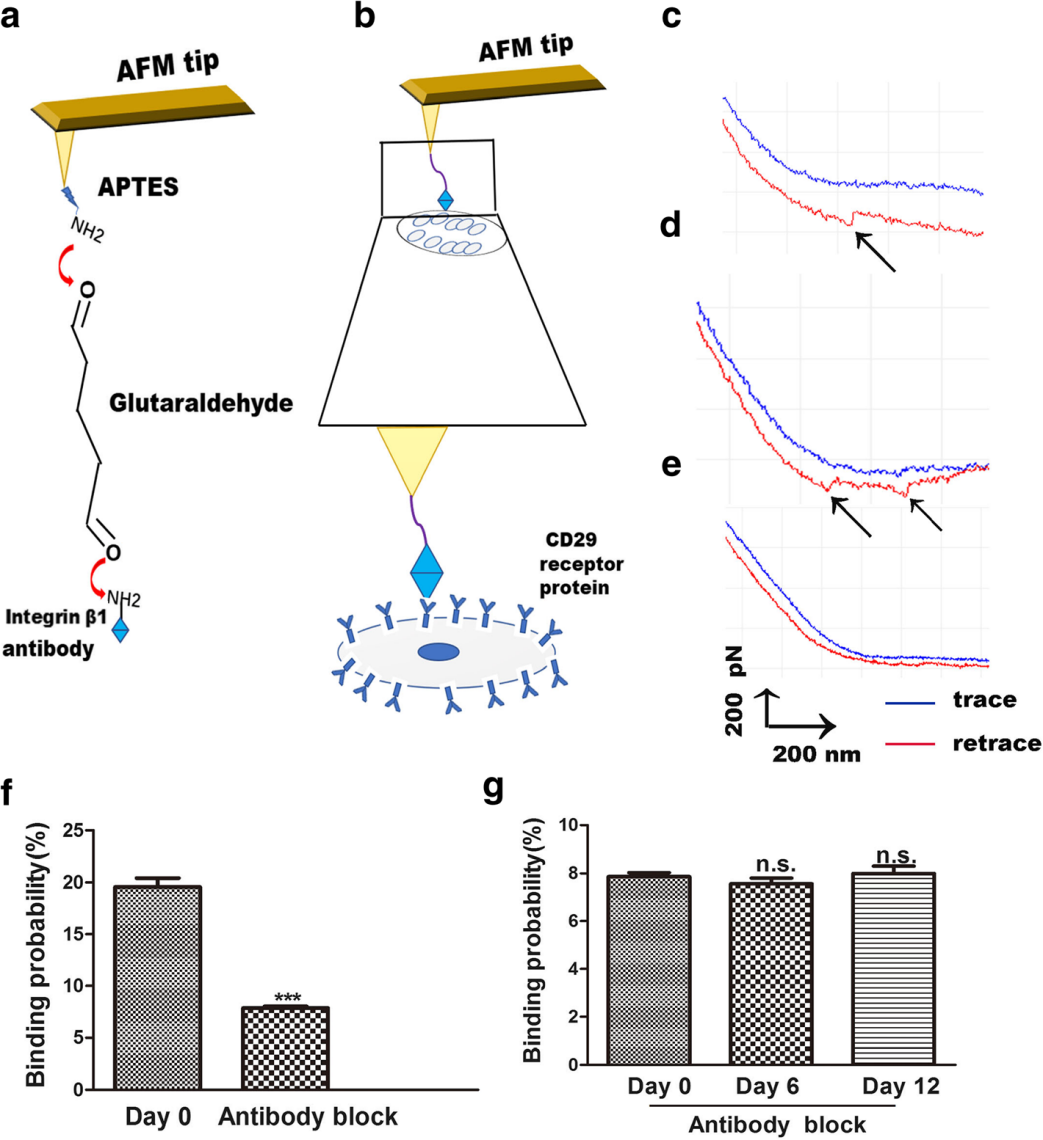
AFM force measurements with integrin β1-functionalized AFM tip on living hADSc. **a** Schematic representation of the strategy used for immobilization of integrin β1 onto an AFM tip. **b** Schematic representation of the single-molecule force measured between integrin β1-functionalized AFM tips and living hADSc. **c, d** Representative force curves obtained with integrin β1-modified AFM tips on hADSc, and **e** after the system was blocked with the integrin β1 monoclonal antibody solution. **f** The binding probability of integrin β1-functionalized tips on hADSc before and after blocking by integrin β1 antibody on day 0. **g** The binding probability of CD29-functionalized tips on hADSc after blocking by integrin β1 antibody at 0, 6, and 12 days. ***p < 0.001, n.s.. no significant difference

AFM is also a useful tool for the study of the binding force between ligands and their receptors, making the membrane-receptor signal transduction on cell surfaces clear [23]. By AFM, changes between integrin β1 and its receptors are measured in a visual, simple, and specific way. The interaction of the integrin β1 ligand–receptor on living cells is a way to explore the binding process on the cell membrane. The procedure for AFM tip functionalization is the coupling of integrin β1 to AFM tips by linkage of APTES and glutaraldehyde. These tips were used for detection of the binding of integrin β1 to CD29 receptors on cell surfaces (Fig. 4a). Single-molecule force spectroscopy (SMFS) was used to assess the anti-integrin β1 living cell separation force distribution within localized regions of individual living hADSc (Fig. 4b). Representative force curves are shown in Fig. 4c, d, which depict a single-molecule curve (Fig. 4c) and two pairs of rupture peak curves (Fig. 4d). Blocking experiments and bare AFM tips experiments were performed to verify the specificity of obtained force curves. Bare AFM tips detected no specific force peak (Fig. 4e). Bare AFM experiments showed that the non-specific binding probability of integrin β1 ligand–receptor interaction on the surface of hADSc was less than 1%. For blocking experiments, the anti-integrin β1 antibody was incubated with cells for 30 min and then force curves were recorded using integrin β1-functionalized tips. The blocking antibody reduced force curves by 90% (Fig. 4f). There was no difference in binding probability of integrin β1 ligand–receptor on cell surfaces among the three groups after anti-integrin β1 antibody treatment (Fig. 4g). These results demonstrate that antibody-modified AFM tips were very useful to detect the force, and that the integrin β1-functionalized AFM tips were specific.

**Fig. 5:**
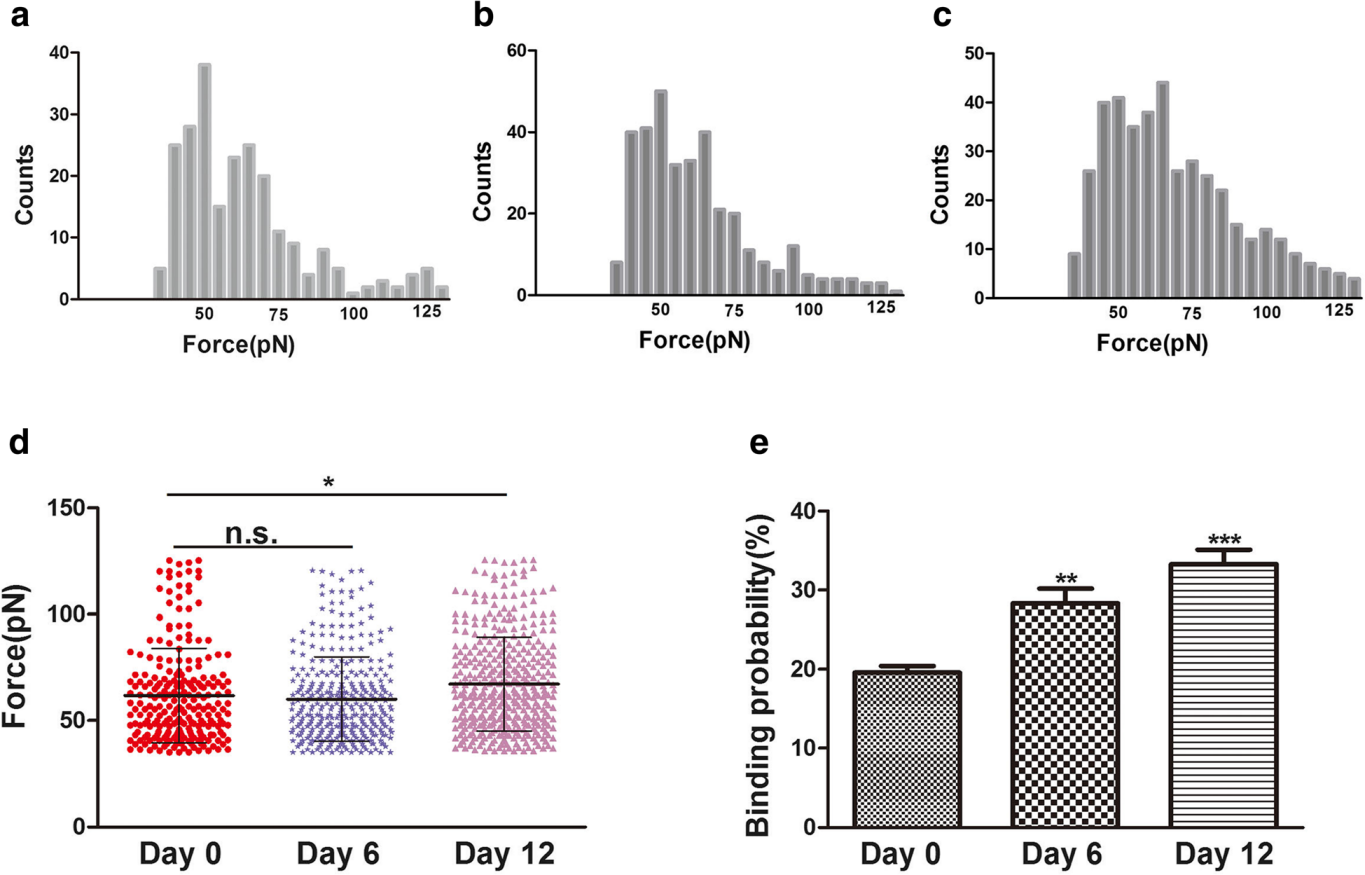
Binding force and binding probability measured on the surface of living hADSc by integrin β1-functionalized AFM tips. **a–c** Histograms of the binding force of integrin β1 antibody–receptor obtained during hADSc chondrogenic differentiation for 0, 6 and 12 days. **d** Binding forces for integrin β1–receptors were obtained on 0, 6, and 12 days of chondrogenic differentiation of hADSc. **e** Binding probability of integrin β1–receptor was detected during chondrogenic differentiation of hADSc for 0, 6, and 12 days. *p < 0.05, **p < 0.01, ***p < 0.001, n.s. no significant difference

The binding force (rupture force) is the interaction force between ligands and their receptors [24]. Changes in morphology and surface ultrastructure of plasma membranes are related to many processes of cellular biology, such as differentiation, apoptosis, and cell migration. During differentiation, changes in the cytoskeleton are thought to be related to integrin changes, especially integrin β1. Integrin β1 (CD29) is very important in cell adhesion to ECM and in cell–cell adhesion. It can also interact with intracellular proteins, stimulating signaling molecules that are related to the actin cytoskeleton [25]. In this study, cytoskeletal and cell morphology changes were observed during hADSc chondrogenesis by confocal laser scanning microscopy (CLSM) and AFM. During chondrogenic differentiation, changes in cytoskeleton, morphology, and surface ultrastructure may be a new and reliable indicator of cell state. Integrin β1, the CD29 receptor, is distributed over the cell surface as judged by immunofluorescence (Fig. 3b). Binding strength and stability of integrin β1 ligand–receptor complexes during hADSc chondrogenesis were evaluated at 0, 6, and 12 days of differentiation. A total of 1200 curves were recorded for each day, with average rupture forces of 61.8 ± 22.2 pN, 60 ± 20.2 pN, and 67.2 ± 22.0 pN, respectively (Fig. 5a–c). The distribution of force magnitude was analyzed as force mean + SD (Fig. 5d). There was no significant difference in force mean between days 0 and 6. There was a difference in force mean between days 0 and 12. The magnitude of the binding force increased at day 12. Meanwhile, rupture events at 0, 6, and 12 days were, respectively, 19.58 ± 1.74%, 28.03 ± 2.05%, and 33.4 ± 1.89% (Fig. 5e). The increased binding probability also indicated that integrin β1 (CD29) played an important role in chondrogenic differentiation and may provide the information for chondrogenic differentiation, via signaling pathways. Hence, increased integrin β1 nanodomains during chondrogenic differentiation may fundamentally affect the binding strength of the CD29 ligand–receptor on living hADSc. Changes in morphology and surface ultrastructure of plasma membranes accompanied changes in integrin β1 protein structure, conformation, binding strength, and stability of integrin β1 ligand–receptor complexes on cells. In summary, integrin β1 plays a necessary role in hADSc chondrogenic differentiation.

### Upregulation of Integrin β1 during hADSc Chondrogenic Differentiation

**Fig. 6:**
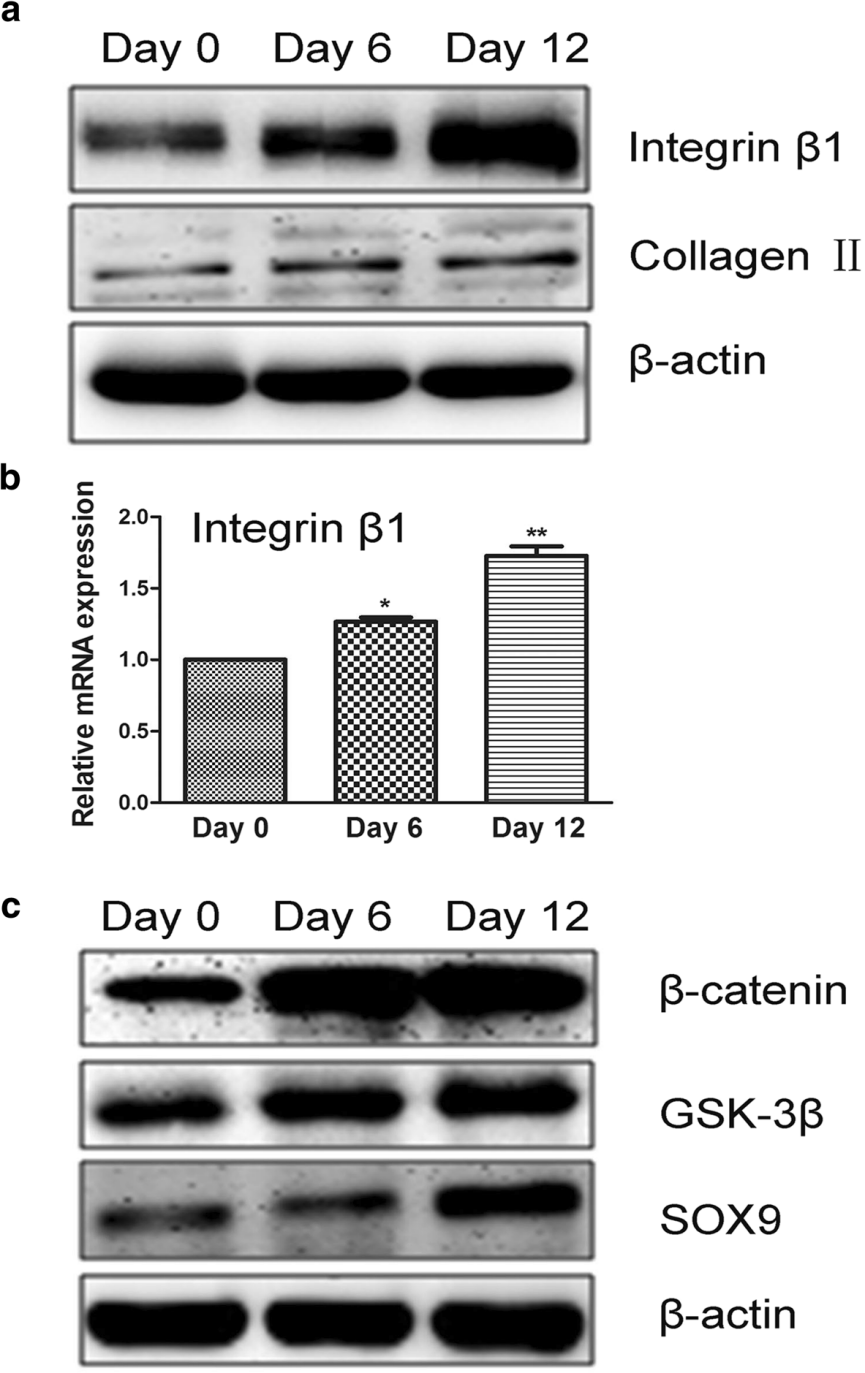
The role of integrin β1 and β-catenin/SOX pathway in regulating hADSc chondrogenic differentiation. **a** Protein integrin β1 was up-regulated during chondrogenesis of hADSc as assessed by western blotting. Cartilage differentiation up-regulated collagen II expression at different days. **b** The mRNA of integrin β1 was up-regulated during chondrogenic differentiation of hADSc. **c** Measurement of proteins associated with the β-catenin/SOX pathway during chondrogenic differentiation of hADSc for 0, 6, and 12 days. *p < 0.05, **p < 0.01

Numerous studies have shown that integrin family members play an important role in cell differentiation. Further, integrins can regulate the interaction between the extracellular environment and cells, controlling signal transduction pathways through connected proteins [26]. Previous studies have shown that binding probability can be affected by the density and conformation of the transmembrane protein (receptors) on the cell surface [27]. Integrin conformation can be a closed headpiece, which has low affinity for ligand, or an open headpiece, which has a high affinity for ligand [28, 29]. Expression of integrin β1 was up-regulated at both the transcriptional and translational levels with increased collagen II expression, characteristic of chondrocytes (Fig. 6a, b). As such, up-regulated integrin β1 expression was consistent with increased binding probability without regard to conformation.

### The Role of Integrin β1 in Chondrogenic Differentiation Regulated by the β-catenin/SOX Signaling Pathway

Previous studies have shown Wnt/β-catenin, PI3K, and mTOR signaling pathways to be related to integrin β1 [30–32]. Each is important in mesenchymal stem cell differentiation. Likewise, studies have demonstrated SOX and collagen II to be regulated by integrin β1 during chondrogenesis of hADSc. SOX is a hallmark component of the Wnt/β-catenin signaling pathway. Hence, we hypothesized that chondrogenic differentiation was regulated by the β-catenin/SOX pathway via integrin β1. SOX, GSK-3β, β-catenin, and integrin β1 were all increased during chondrogenesis of hADSc (Fig. 6c), with integrin β1 inducing cell signaling. These data demonstrate chondrogenic differentiation to be regulated by the β-catenin/SOX pathway via integrin β1.

### Prospective and Limitations

In this work, changes in cellular morphology, the structure of the membrane, and the binding probability of integrin β1 ligand–receptors were demonstrated to be useful image markers to evaluate the chondrogenic differentiation process. This is a new method for evaluation of morphology, membrane ultrastructure, and changes in transmembrane proteins during chondrogenic differentiation. There are limitations to this study. Although increased binding probability was related to the high expression of integrin β1, the conformation of integrin β1 during chondrogenesis was not investigated. Further work is necessary to determine the conformation of integrin β1 during chondrogenic differentiation. Integrin β1 was demonstrated to participate in the β-catenin/SOX signaling pathway during chondrogenesis of hADSc. However, the relationship between integrin β1 and β-catenin/SOX signaling pathway is still not fully established. Further work is necessary to identify the exact role of integrin β1 in this pathway.

## Conclusions

In the present work, a novel method (AFM) was employed to evaluate chondrogenic induction in hADSc. Cell surface ultrastructural changes were assessed by AFM imaging. AFM was used to investigate the binding force and binding probability between integrin β1 ligand and its receptors on the surface of hADSc by integrin β1-functionalized AFM tips. Based on AFM data, during chondrogenesis, cell morphology was changed from an elongated spindle shape to a polygonal shape with increased cell roughness. By use of integrin β1-functionalized AFM tips, the binding probability and force magnitude of integrin β1 ligand–receptor on the surface of hADSc were found to increase during chondrogenic induction. By immunoblot, integrin β1 was demonstrated to participate in the β-catenin/SOX signaling pathway, which regulated the chondrogenesis of hADSc. Taken together, these results and the established methodology contribute to a better understanding of cell morphology and roughness. Further, the data provide thermodynamic and kinetic insight into the integrin β1 ligand-binding process, at the single-molecule level. This AFM method will be useful for investigation of signaling pathways in living hADSc during chondrogenesis. Changes in the cellular nanostructure, as well as structure of the membrane, and the binding probability of transmembrane proteins are useful markers to evaluate chondrogenic differentiation mechanisms. This AFM method can be used to understand the mechanism of mesenchymal stem cell differentiation in tissue engineering and will be useful for an enhanced understanding of mesenchymal stem cell chondrogenic differentiation.

## Additional files

Additional file 1: **Figure S1.** The surface antigens of hADSc detected by flow cytometry. (JPG 528 kb)

Additional file 2: **Figure S2.** Representative images of hADSc after differentiation. **a, b** Representative images of hADSc with Alcian Blue staining and Toluidine Blue staining, respectively, indicating chondrogenic differentiation. **c** Representative images of hADSc with Alizarin Red staining, indicating osteogenic differentiation. **d** Representative images of hADSc with Oil Red O staining, indicating adipogenic differentiation. All the pictures were magnified 100 times by microscope. (JPG 3076 kb)

Additional file 3: **Figure S3.** The hADSc proliferation curve. (JPG 40 kb)

## Abbreviations

AFM: Atomic force microscopy
CD: Cluster of differentiation
DAPI: 4′,6-diamidino-2-phenylindole
DMEM: Dulbecco’s Modified Eagle’s Medium
ECM: Extracellular matrix
F-actin: Filamentous actin
FBS: Fetal bovine serum
hADSc: Human adipose-deprived stem cells
IB: Immunoblotting analysis
IBMX: 3-isobutyl-1-methylxanthine
IGF-1: Insulin-like growth factors-1
ITS: Insulin transferrin selenium
mTOR: Mammalian target of rapamycin
OA: Osteoarthritis
PBS: Phosphate buffer solution
PI3K: Phosphoinositide 3-kinase
SD: Standard deviation
SMFS: Single-molecule force spectroscopy
TGF-β1: Transforming growth factor-beta1

## References

[1] Cross M, Smith E, Hoy D, Nolte S, Ackerman I, Fransen M (2013). The global burden of hip and knee osteoarthritis: estimates from the global burden of disease 2010 study. Ann Rheum Dis.

[2] Becerra J, Andrades JA, Guerado E, Zamora-Navas P, López-Puertas JM, Reddi AH (2010). Articular cartilage: structure and regeneration. Tissue Eng B Rev.

[3] Taruc-Uy RL, Lynch SA (2013). Diagnosis and treatment of osteoarthritis. Prim Care.

[4] Makris EA, Gomoll AH, Malizos KN, Hu JC, Athanasiou KA (2015). Repair and tissue engineering techniques for articular cartilage. Nat Rev Rheumatol.

[5] Smith A, Marquis M, Vinatier C, Rieux AD, Renard D, Guicheux J (2017). Mesenchymal stem cells-containing alginate particles for intra-articular injection in osteoarthritis. Osteoarthr Cartil.

[6] Xia P, Wang X, Qu Y, Lin Q, Cheng K, Gao M (2017). TGF-beta1-induced chondrogenesis of bone marrow mesenchymal stem cells is promoted by low-intensity pulsed ultrasound through the integrin-mTOR signaling pathway. Stem Cell Res Ther.

[7] Ozeki N, Mogi M, Hase N, Hiyama T, Yamaguchi H, Kawai R (2016). Autophagy-related gene 5 and Wnt5 signaling pathway requires differentiation of embryonic stem cells into odontoblast-like cells. Exp Cell Res.

[8] Lee JS, Yi JK, An SY, Heo JS (2014). Increased osteogenic differentiation of periodontal ligament stem cells on polydopamine film occurs via activation of integrin and PI3K signaling pathways. Cell Physiol Biochem.

[9] Campbell ID, Humphries MJ (2011). Integrin structure, activation, and interactions. Cold Spring Harb Perspect Biol.

[10] Barczyk M, Carracedo S, Gullberg D (2010). Integrins. Cell Tissue Res.

[11] Meghana C, Ramdas N, Hameed FM, Rao M, Shivashankar GV, Narasimha M (2011). Integrin adhesion drives the emergent polarization of active cytoskeletal stresses to pattern cell delamination. Proc Natl Acad Sci U S A.

[12] Parekh R, Lorenzo MK, Shin SY, Pozzi A, Clark AL (2014). Integrin α1β1 differentially regulates cytokine-mediated responses in chondrocytes. Osteoarthr Cartil.

[13] Luo S, Shi Q, Zha Z, Ping Y, Lin H, Ning L (2012). Morphology and mechanics of chondroid cells from human adipose-derived stem cells detected by atomic force microscopy. Mol Cell Biochem.

[14] Zhang HT, Yang J, Liang GH, Gao XJ, Sang Y, Gui T (2017). Andrographolide induces cell cycle arrest and apoptosis of chondrosarcoma by targeting TCF-1/SOX9 axis. J Cell Biochem.

[15] Pi J, Cai H, Yang F, Jin H, Liu J, Yang P (2016). Atomic force microscopy based investigations of anti-inflammatory effects in lipopolysaccharide-stimulated macrophages. Anal Bioanal Chem.

[16] Burrow KL, Hoyland JA, Richardson SM (2017). Human adipose-derived stem cells exhibit enhanced proliferative capacity and retain multipotency longer than donor-matched bone marrow mesenchymal stem cells during expansion in vitro. Stem Cells Int.

[17] Dufrãªne YF, Ando T, Garcia R, Alsteens D, Martinez-Martin D, Engel A (2017). Imaging modes of atomic force microscopy for application in molecular and cell biology. Nat Nanotechnol.

[18] Huang X, He J, Zhang H, Sun K, Yang J, Wang H (2017). Effect of dacarbazine on CD44 in live melanoma cells as measured by atomic force microscopy-based nanoscopy. Int J Nanomedicine.

[19] Ziperstein MJ, Guzman A, Kaufman LJ (2016). Evaluating breast Cancer cell morphology as a predictor of invasive capacity. Biophys J.

[20] Tsimbouri P, Gadegaard N, Burgess K, White K, Reynolds P, Herzyk P (2014). Nanotopographical effects on mesenchymal stem cell morphology and phenotype. J Cell Biochem.

[21] McBeath R, Pirone DM, Nelson CM, Bhadriraju K, Chen CS (2004). Cell shape, cytoskeletal tension, and RhoA regulate stem cell lineage commitment. Dev Cell.

[22] Girasole M, Pompeo G, Cricenti A, Congiu-Castellano A, Andreola F, Serafino A (2007). Roughness of the plasma membrane as an independent morphological parameter to study RBCs: a quantitative atomic force microscopy investigation. Biochim Biophys Acta.

[23] Shan Y, Wang H (2015). The structure and function of cell membranes examined by atomic force microscopy and single-molecule force spectroscopy. Chem Soc Rev.

[24] Whited AM, Park PS-H (2014). Atomic force microscopy: a multifaceted tool to study membrane proteins and their interactions with ligands. Biochimica et Biophysica Acta (BBA)-Biomembranes.

[25] Walther CG, Whitfield R, James DC (2016). Importance of interaction between integrin and actin cytoskeleton in suspension adaptation of CHO cells. Appl Biochem Biotechnol.

[26] Marie PJ (2013). Targeting integrins to promote bone formation and repair. Nat Rev Endocrinol.

[27] Friedsam C, Bécares ADC, Jonas U, Gaub HE, Seitz M (2004). Polymer functionalized AFM tips for long-term measurements in single-molecule force spectroscopy. Chem Phys Chem.

[28] Luo BH, Carman CV, Springer TA (2007). Structural basis of integrin regulation and signaling. Annu Rev Immunol.

[29] Mcever RP, Zhu C (2007). A catch to integrin activation. Nat Immunol.

[30] Hill TP, Später D, Taketo MM, Birchmeier W, Hartmann C (2005). Canonical Wnt/β-catenin signaling prevents osteoblasts from differentiating into chondrocytes. Dev Cell.

[31] Lian I, Kim J, Okazawa H, Zhao J, Zhao B, Yu J (2010). The role of YAP transcription coactivator in regulating stem cell self-renewal and differentiation. Genes Dev.

[32] Phornphutkul C, Wu KY, Auyeung V, Chen Q, Gruppuso PA (2008). mTOR signaling contributes to chondrocyte differentiation. Dev Dyn.

