# Supplementary figures and images for: Atomic Force Microscopy-Based Nanoscopy of Chondrogenically Differentiating Human Adipose-Derived Stem Cells: Nanostructure and Integrin β1 Expression
Source: Nanoscale Res Lett. 2018 Oct 23;13:333. doi: 10.1186/s11671-018-2722-z (PMC6199198; doi:10.1186/s11671-018-2722-z)

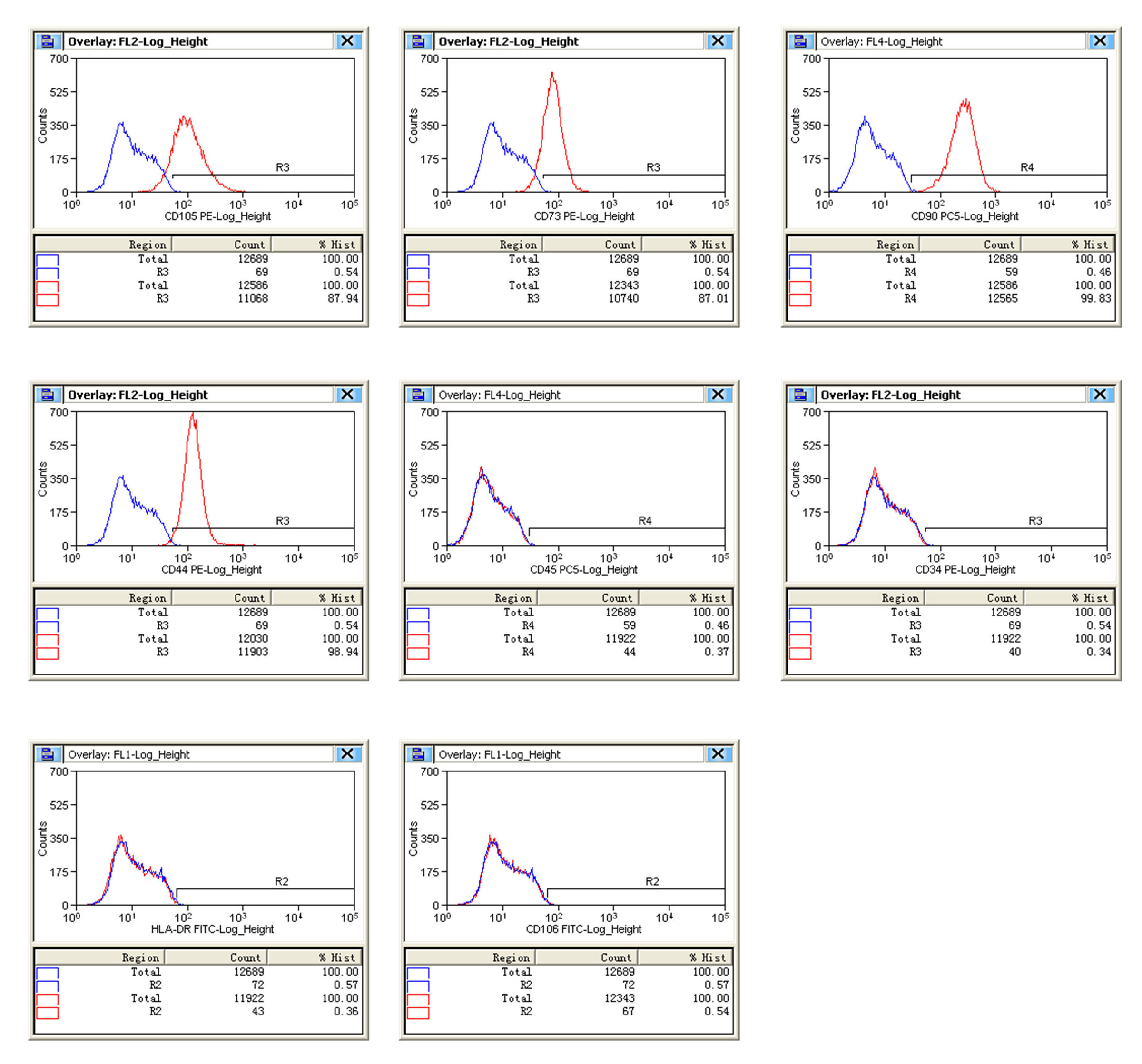

Supplement: Supplementary file 1 — Figure S1. The surface antigens of hADSc detected by flow cytometry. (JPG 528 kb) [file 11671_2018_2722_MOESM1_ESM.jpg]

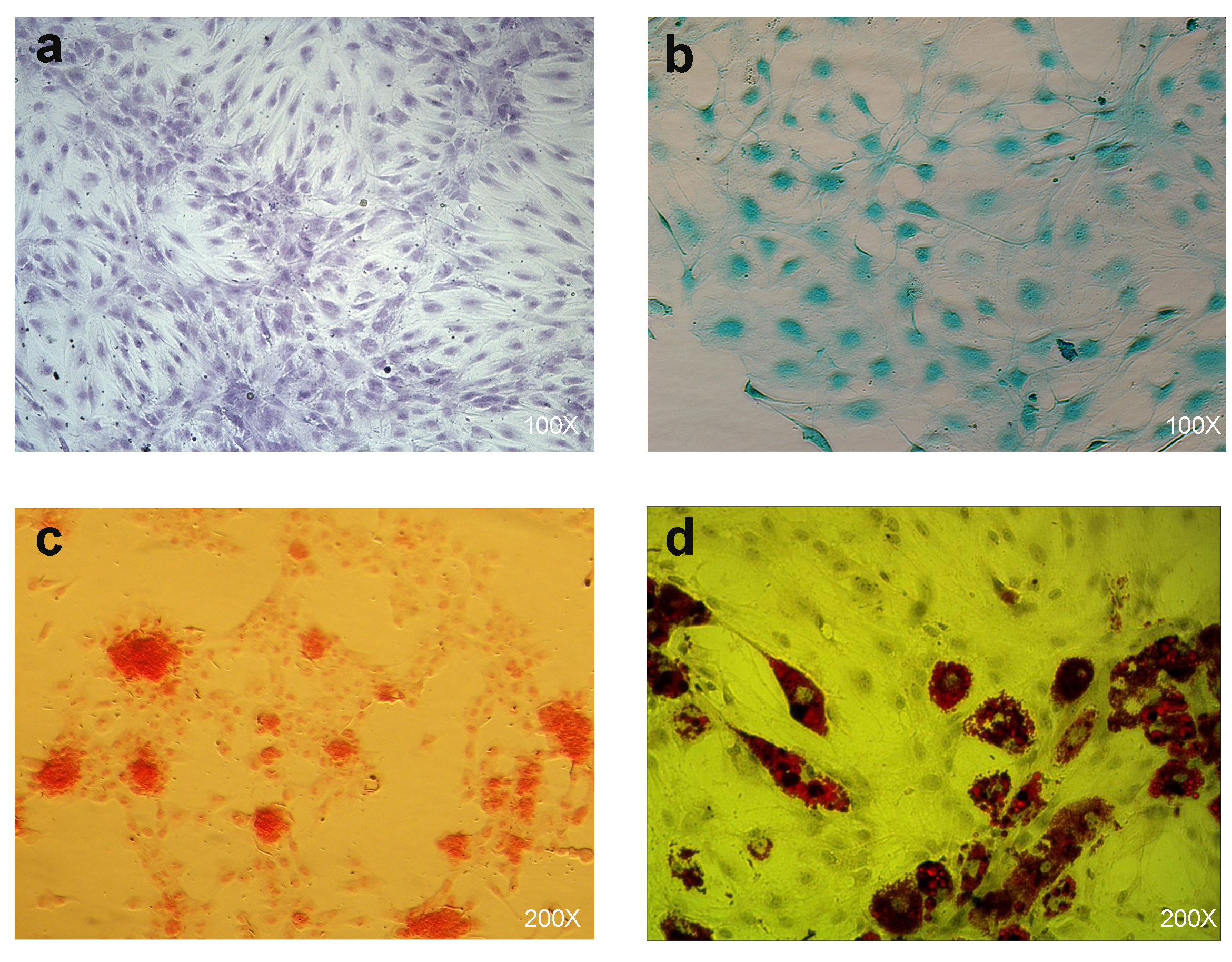

Supplement: Supplementary file 2 — Figure S2. Representative images of hADSc after differentiation. a, b Representative images of hADSc with Alcian Blue staining and Toluidine Blue staining, respectively, indicating chondrogenic differentiation. c Representative images of hADSc with Alizarin Red staining, indicating osteogenic differentiation. d Representative images of hADSc with Oil Red O staining, indicating adipogenic differentiation. All the pictures were magnified 100 times by microscope. (JPG 3076 kb) [file 11671_2018_2722_MOESM2_ESM.jpg]

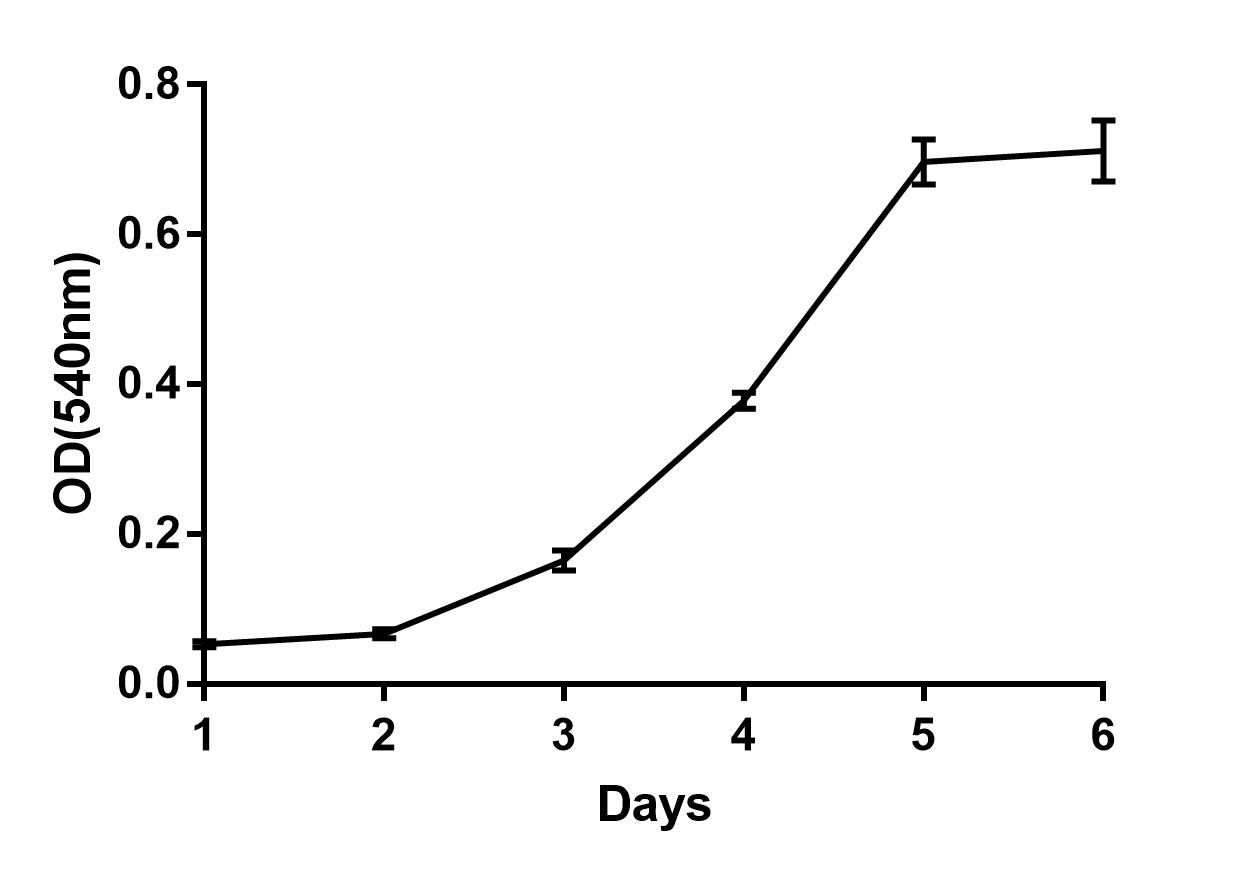

Supplement: Supplementary file 3 — Figure S3. The hADSc proliferation curve. (JPG 40 kb) [file 11671_2018_2722_MOESM3_ESM.jpg]
